# Reduced type I interferon production by dendritic cells and weakened antiviral immunity in patients with Wiskott-Aldrich syndrome protein deficiency

**DOI:** 10.1016/j.jaci.2012.08.050

**Published:** 2013-03

**Authors:** Philipp A. Lang, Namir Shaabani, Stephanie Borkens, Nadine Honke, Stefanie Scheu, Sarah Booth, Dirk Brenner, Andreas Meryk, Carmen Barthuber, Mike Recher, Tak W. Mak, Pamela S. Ohashi, Dieter Häussinger, Gillian M. Griffiths, Adrian J. Thrasher, Gerben Bouma, Karl S. Lang

**Affiliations:** a Campbell Family Institute for Breast Cancer Research, Ontario Cancer Institute, Toronto, Ontario, Canada; b Department of Gastroenterology, Hepatology and Infectious Diseases, University of Düsseldorf, Düsseldorf, Germany; d Institute of Medical Microbiology and Hospital Hygiene, University of Düsseldorf, Düsseldorf, Germany; f Department of Laboratory Medicine, University of Düsseldorf, Düsseldorf, Germany; c Institute for Immunology, University of Essen, Essen, Germany; e Sir William Dunn School of Pathology, Oxford, United Kingdom; g Clinical Immunology, University Hospital, Basel, Switzerland; h Molecular Immunology Unit, Institute of Child Health, University College London, London, United Kingdom; i Great Ormond Street Hospital NHS Foundation Trust, London, United Kingdom

**Keywords:** Type I interferon, dendritic cells, CD8 T cells, virus, Wiskott-Aldrich syndrome protein, Wiskott-Aldrich syndrome, diabetes, cDC, Conventional dendritic cell, DC, Dendritic cell, FACS, Fluorescence-activated cell sorting, IFN-I, Type I interferon, IL-7R, IL-7 receptor, LCMV, Lymphocytic choriomeningitis virus, NK, Natural killer, pDC, Plasmacytoid dendritic cell, Poly(I:C), Polyinosine-polycytidylic acid, pfu, Plaque-forming units, TLR, Toll-like receptor, VSV, Vesicular stomatitis virus, WAS, Wiskott-Aldrich syndrome, WAS KO, WASP knockout, WASP, Wiskott-Aldrich syndrome protein, YFP, Yellow fluorescent protein

## Abstract

**Background:**

Wiskott-Aldrich syndrome (WAS) is a rare X-linked primary immunodeficiency caused by absence of Wiskott-Aldrich syndrome protein (WASP) expression, resulting in defective function of many immune cell lineages and susceptibility to severe bacterial, viral, and fungal infections. Despite a significant proportion of patients with WAS having recurrent viral infections, surprisingly little is known about the effects of WASP deficiency on antiviral immunity.

**Objective:**

We sought to evaluate the antiviral immune response in patients with WASP deficiency *in vivo*.

**Methods:**

Viral clearance and associated immunopathology were measured after infection of WASP-deficient (WAS KO) mice with lymphocytic choriomeningitis virus (LCMV). Induction of antiviral CD8^+^ T-cell immunity and cytotoxicity was documented in WAS KO mice by means of temporal enumeration of total and antigen-specific T-cell numbers. Type I interferon (IFN-I) production was measured in serum in response to LCMV challenge and characterized *in vivo* by using IFN-I reporter mice crossed with WAS KO mice.

**Results:**

WAS KO mice showed reduced viral clearance and enhanced immunopathology during LCMV infection. This was attributed to both an intrinsic CD8^+^ T-cell defect and defective priming of CD8^+^ T cells by dendritic cells (DCs). IFN-I production by WAS KO DCs was reduced both *in vivo* and *in vitro*.

**Conclusions:**

These studies use a well-characterized model of persistence-prone viral infection to reveal a critical deficiency of CD8^+^ T-cell responses in murine WASP deficiency, in which abrogated production of IFN-I by DCs might play an important contributory role. These findings might help us to understand the immunodeficiency of WAS.

Wiskott-Aldrich syndrome (WAS) is a rare X-linked genetic human disease associated with thrombocytopenia, eczema, and life-threatening immunodeficiency.^1,2^ Patients often also have an increased incidence of autoimmune disease and malignancies.^3,4^ WAS is caused by mutations in the gene encoding the Wiskott-Aldrich syndrome protein (WASP), which is a member of a family of proteins that are required for the transduction of signals from the cell surface to the actin cytoskeleton.^5^ Because expression of WASP is restricted to cells of the hematopoietic lineage, the absence of WASP results in defective function of many immune cell lineages, leading to a combined cellular and humoral immune defect. Defective immune cell function resulting from WASP deficiency is multifactorial, including global defects of migration of lymphoid and myeloid lineages,^6-10^ as well as impaired cell-specific effector function. For instance, in the absence of WASP function, uptake of particulate antigen by macrophages by means of phagocytosis is defective,^6-12^ podosome formation and T-cell priming ability of dendritic cells (DCs) is impaired,^6,13-15^ B- and T-cell proliferation in response to B- or T-cell receptor ligation is reduced,^16-20^ and homeostasis of mature B-cell populations,^21,22^ as well as homeostasis and function of regulatory T cells, is disturbed.^23-26^ Therefore the severe immunodeficiency resulting from WASP deficiency is thought to be the result of a complex combination of cellular immune defects.

Although many studies have focused on characterizing the role of WASP in individual immune cell lineages, much is unknown about the role of WASP for antiviral immunity despite a significant proportion of patients with WAS having recurrent infections, most commonly involving members of the herpes virus family.^3,4^ Previous reports have indicated susceptibility of WAS KO mouse models to influenza infection, which seemed more pronounced after secondary viral challenge, reflecting impaired memory function.^27,28^ Recently, WASP-deficient CD8^+^ cells derived from patients have been shown to polarize lytic granules poorly and to exhibit diminished cytotoxicity.^29^ Acute and chronic infection with persistence-prone viruses often results in organ-specific immunopathology.^30-32^ In this setting virus-specific T cells are a major determinant of immunopathology because they contribute to organ infiltration and are directly cytotoxic to virus-infected target cells.^33^ Mechanisms of virus control and virus-induced immunopathology have been studied in mice by using the noncytopathic RNA virus lymphocytic choriomeningitis virus (LCMV), in which immunopathology is predominantly mediated by CD8^+^ T cell–mediated cytotoxicity against virus-infected cells. Complete lack of CD8^+^ T cells strongly reduces immunopathology, even though viral replication is enhanced.^34^ In contrast, delayed innate or adaptive immune response also enhances viral replication in the target organ, leading to exaggerated immunopathology.^33,35^ In addition to CD8^+^ T cells, type I interferon (IFN-I) is crucial for the control of viral replication^36^ and induced at early time points after viral infection. Host production of IFN-I is elicited by the ligation of host pattern-recognition receptors by viral molecules, generally activating the transcription factor interferon regulatory factor 7, which then translocates to the nucleus and promotes IFN-I production.^37^

Here we have used models of virus-induced immunopathology to analyze antiviral immunity in patients with WASP deficiency. We found that WASP deficiency results in impaired viral clearance and enhanced immunopathology. This was associated with an impaired CD8^+^ T-cell response and reduced production of IFN-Is by DCs.

## Methods

### Mice and viruses

WAS KO, RIP-GP, and IFNβ^mob/mob^ mice were bred in our own facilities. Control C57BL/6 mice were purchased from Charles River (Margate, United Kingdom). For some experiments, WAS KO mice were crossed with IFNβ^mob/mob^ mice (both on a C57BL/6 background). WAS KO mice were also crossed to RIP-GP mice (on a C57BL/6 background). For generation of WAS KO bone marrow chimeras in RIP-GP mice (both on a C57BL/6 background), recipient mice were irradiated with 1050 rad on day −1. On day 0, 10^7^ bone marrow cells were transferred intravenously, and mice were used for experiments 7 weeks later. All experiments were performed in single ventilated cages. Animal experiments were carried out either with authorization of the Veterinäramt of the Kanton Zurich and in accordance with the Swiss law for animal protection or with the approval of and according to United Kingdom Home Office Animal Welfare legislation. Further animal experiments were carried out with the authorization of the Landesamt für Natur, Umwelt und Verbraucherschutz of Nordrhein-Westfalen, Germany, and in accordance with the German law for animal protection, the institutional guidelines of the Ontario Cancer Institute, or both.

LCMV strain WE was originally obtained from F. Lehmann-Grube (Heinrich Pette Institute, Hamburg, Germany) and propagated in L929 cells. Viral titers were measured by using a plaque-forming assay, as previously described.^38^ Mice were infected with 200 plaque-forming units (pfu) of LCMV-WE unless stated otherwise. Vesicular stomatitis virus (VSV), Indiana strain (VSV-IND, Mudd-Summers isolate), was originally obtained from Professor D. Kolakofsky (University of Geneva, Geneva, Switzerland). Virus was propagated on BHK-21 cells at a multiplicity of infection of 0.01 followed by plaquing onto Vero cells.

### Immunohistochemistry

Cryostat sections of 7 μm in thickness were cut onto poly-l-lysine–coated slides (VWR, Leuven, Belgium), fixed for 20 minutes in 1% paraformaldehyde (BDH, Poole, United Kingdom), and rinsed in PBS. Slides were blocked with 2% normal mouse serum (Dako Cytomation, Glostrup, Denmark), followed by incubation for at least 1 hour with primary antibodies specific for CD4 (eBioscience, San Diego, Calif), CD8 (BD Biosciences, San Jose, Calif), or MHC I (BioLegend, San Diego, Calif). After washing, slides were incubated for 30 to 45 minutes with alkaline phosphatase–conjugated anti-rat secondary antibody (Dako). Naphthol red was used as a substrate (Sigma, St Louis, Mo), and hematoxylin (Merck, Darmstadt, Germany) was used for counterstaining.

### Assessment of diabetes

Blood glucose concentrations were analyzed from a drop of blood by using a Glucometer Elite (Bayer, Tarrytown, NY). When animals showed blood glucose levels of greater than 14 mmol/L on 2 consecutive days, they were considered diabetic.

### Fluorescence-activated cell sorting analysis

Tetramer production and fluorescence-activated cell sorting (FACS) analysis were performed as described previously.^39^ Briefly, splenocytes or peripheral blood lymphocytes were stained with phycoerythrin-labeled GP33 MHC class I tetramers (GP33/H-2D^b^) for 15 minutes at 37°C, followed by staining with anti-CD8 (BD Biosciences) for 30 minutes at 4°C. For determination of LCMV-specific CD4 T cells, lymphocytes were stained with anti-CD4 and anti-Thy1.1 (CD90.1, BD Biosciences). For determination of their activation status, lymphocytes were stained with anti-CD25, anti-CD69, anti-GITR, anti-CD62 ligand, anti-CD44, and anti–IL-7 receptor α (BD Biosciences) for 30 minutes at 4°C. Cells were fixed with 1% formalin and permeabilized with saponin. Cells were stained for intracellular IFN-γ, IL-10, IL-4 (BD Biosciences), and intracellular Granzyme B (CALTAG, Burlingame, Calif). IFN-α production by DCs was assessed by means of intracellular FACS after culture of bone marrow cells for 10 days with Flt3L (100 ng/mL; Peprotech, Rocky Hills, NJ). For identification of DCs, CD11c (BD Biosciences) was used, and CD8α, CD11b, and B220 or pDCA1 (BD Biosciences) were used to distinguish between CD8^+^ cells, conventional dendritic cells (cDCs), and plasmacytoid dendritic cells (pDCs), respectively. Cells were analyzed with a FACSCalibur or FACSCanto II (BD Biosciences).

### T-cell priming

DCs were cultured from bone marrow cells in the presence of GM-CSF (20 ng/mL; Invitrogen, Carlsbad, Calif) for 7 days and pulsed overnight with ovalbumin (100 μg/mL, Sigma) and LPS (100 ng/mL, Sigma). DCs (2 × 10^6^) were injected subcutaneously into the tail base of wild-type C57BL/6 mice, and spleens and draining lymph nodes (inguinal) were harvested at the indicated time points. Single-cell suspensions of lymph nodes and spleens were cocultured with SIINFEKL peptide (2 μmol/L; Proimmune, Oxford, United Kingdom) and RMA-S cells (kindly provided by Dr Anne-Marie McNicol) in the presence of brefeldin A (5 μg/mL, Sigma). The RMA-S cells were incubated overnight at 26°C before the experiment to establish expression of empty MHC class I molecules on the cell surface, which will present the SIINFEKL peptide during culture with lymph node or spleen cells. After 4 hours, the cells were stained with fluorescein isothiocyanate (FITC)–conjugated CD3 and peridinin-chlorophyll-protein complex–conjugated CD8 and then permeabilized (BD Perm/Wash, BD PharMingen) and stained with phycoerythrin-conjugated IFN-γ (eBioscience). The cells were analyzed on a Cyan flow cytometer.

### IFN-α ELISA

Mice were infected with LCMV or VSV or injected with polyinosine-polycytidylic acid (Poly[I:C]) and blood obtained at the indicated time points. Serum IFN-α levels were determined by using ELISA, according to the manufacturers' specifications (Research Diagnostics RDI, Flanders, NJ).

### Cytotoxicity assay

EL4 target cells were loaded with ^51^Cr and pulsed with or without GP33 or NP396. Splenocytes of immunized mice were incubated directly *ex vivo* (primary) or after restimulation with GP33 or NP396 for 5 days (secondary) with the target cells. Supernatants were assessed after 8 hours. For killing of allogeneic BALB/c splenocytes, a Cytotox 96 nonradioactive kit (Promega, Madison, Wis) was used according to the instructions provided. Ficoll-purified T cells were plated at the effector/target ratios shown by using 10^4^ BALB/c splenocytes (target cells). Lactate dehydrogenase release was assayed after 4 hours of incubation at 37°C.

Percentage cytotoxicity = (Experimental effector spontaneous − Target spontaneous/Target maximum − Target spontaneous) × 100.

### Statistical analysis

Data are expressed as means ± SEMs. When comparing data expressed as curves, linear regression was used. When curves did not follow a linear pattern, the area under the curve or peak values were determined and compared by using the Student *t* test. When comparing 2 groups, the Student *t* test was used. Survival data were analyzed by using the log rank test. All statistical tests were performed with Prism 5 software (GraphPad Software, La Jolla, Calif). *P* values of less than .05 were considered statistically significant.

## Results

### Reduced viral clearance in WAS KO mice

**Fig 1:**
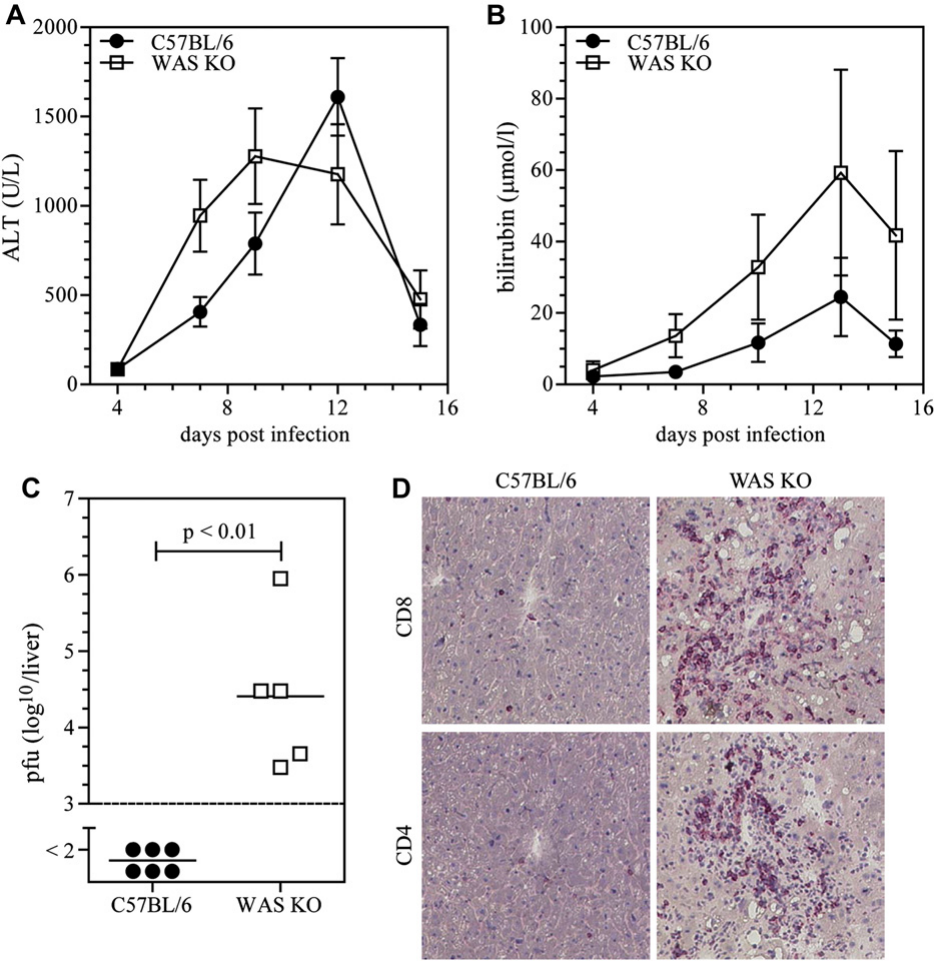
Absence of WASP enhances virus-induced immunopathology. Virus-induced immunopathology was analyzed as serum alanine aminotransferase *(ALT)* activity **(A)** and bilirubin levels **(B)** after LCMV infection. Viral clearance was assessed by measuring viral titers in the liver **(C)**. The presence of CD4^+^ and CD8^+^ T cells in the liver was analyzed by means of immunohistochemistry **(D)**. Data in Fig 1, *A* and *B*, are means ± SEMs and peaks (Fig 1, *A*: C57BL/6 mice, n = 6-12; WAS KO mice, n = 8-10) or areas under the curve (Fig 1, *B*: C57BL/6 mice, n = 5-9; WAS KO mice, n = 5-6) compared by using the Student *t* test. Symbols in Fig 1, *C*, represent individual mice, and images in Fig 1, *D*, are representative of data shown in Fig 1, *C*.

To investigate the ability of WAS KO mice to mount a protective immune response against viral infection and to investigate immunopathology, we challenged mice with LCMV (WE strain). Serum alanine aminotransferase and total bilirubin levels were used as a direct measurement of virus-induced hepatic immunopathology.^33^ WAS KO mice showed a similar, although slightly earlier, alanine aminotransferase response but exhibited increased bilirubin levels after viral challenge (Fig 1, *A* and *B*). Furthermore, WAS KO mice showed persistence of viral replication and a hepatic T-cell infiltrate 15 days after infection, whereas by that time, wild-type C57BL/6 animals had cleared viral infection and showed no sign of T-cell infiltration (Fig 1, *C* and *D*). These finding indicate that WAS KO mice are compromised in their ability to clear LCMV, despite the presence of an inflammatory T-cell infiltrate.

### Defective induction of CD8^+^ T-cell responses

**Fig 2:**
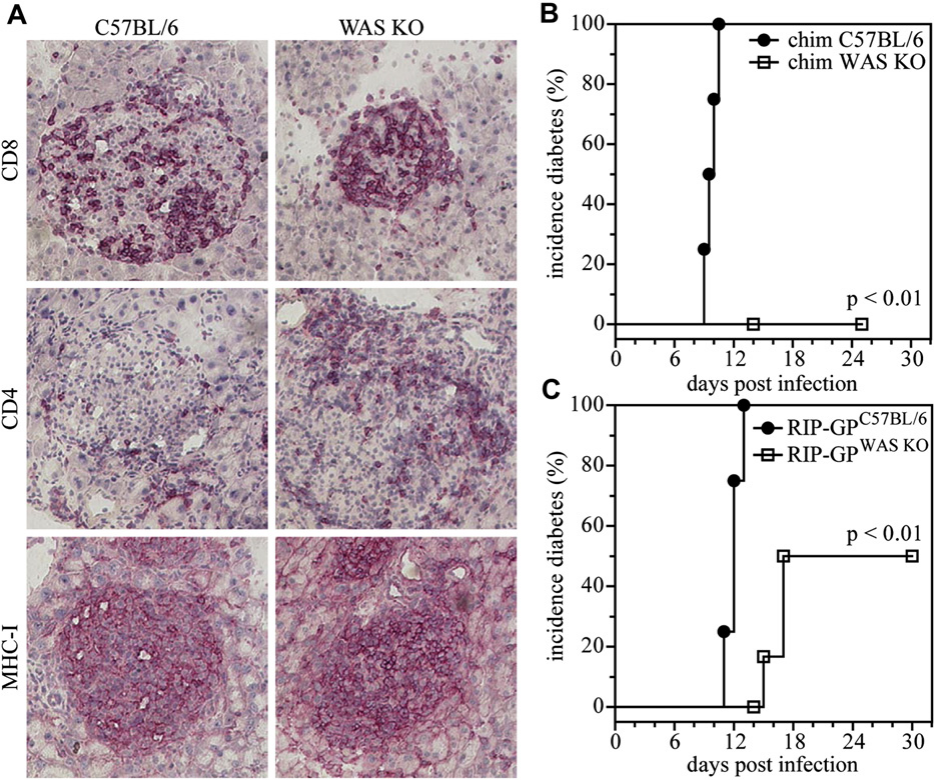
Reduced incidence of virus-induced diabetes. C57BL/6 and WAS KO bone marrow was transferred into irradiated RIP-GP mice, and after 50 days, mice were infected with LCMV. Insulitis was determined by means of immunohistochemistry **(A)** and incidence of diabetes analyzed (**B**; n = 4). Transgenic WAS KO/RIP-GP mice were made by crossing RIP-GP mice with WAS KO mice. Mice were infected with LCMV, and the incidence of diabetes was analyzed (**C**; n = 8).

We used the RIP-GP model of autoimmune diabetes to study the induction of an effective antiviral CD8^+^ T-cell response *in vivo*. These mice express the LCMV glycoprotein under regulatorycontrol of the rat insulin promoter, resulting in β cell–restricted expression. On infection with LCMV, healthy mice mount an anti-LCMV immune response, which is dominated by the generation of glycoprotein-specific cytotoxic CD8^+^ T cells that not only clear the virus but also destroy the β cells that express the LCMV glycoprotein, subsequently inducing development of diabetes.^40^ We created bone marrow chimeras by transferring WAS KO or wild-type C57BL/6 bone marrow into lethally irradiated RIP-GP mice and challenged the mice after a further 50 days with LCMV. Despite the development of insulitis (Fig 2, *A*), characterized by infiltration of CD4^+^ and CD8^+^ T cells into the β-cell–containing islets of Langerhans, and generalized upregulated expression of MHC class I as a result of inflammatory conditions, mice reconstituted with WAS KO bone marrow did not have overt diabetes (Fig 2, *B*). Similarly, when RIP-GP mice were crossed with WAS KO mice, the incidence of overt diabetes after LCMV challenge was significantly reduced compared with that seen in RIP-GP single transgenic animals (Fig 2, *C*), although not to the extent seen in bone marrow chimeric mice, which is probably due to the additional immunosuppressive effects of irradiation and bone marrow transplantation.

**Fig E1:**
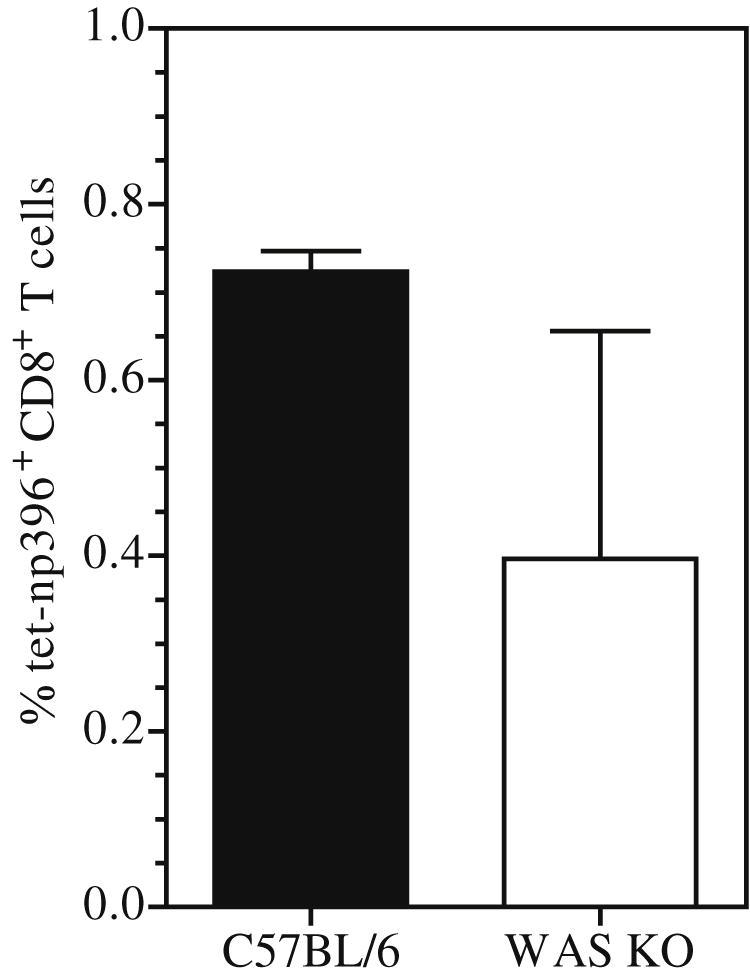
Analysis of the T-cell response for the immunodominant epitope of LCMV nucleoprotein. The virus-specific CD8^+^ T-cell response for the immunodominant epitope of the LCMV nucleoprotein was analyzed 12 days after LCMV infection. Data are shown as means ± SEMs (n = 3).

**Fig 3:**
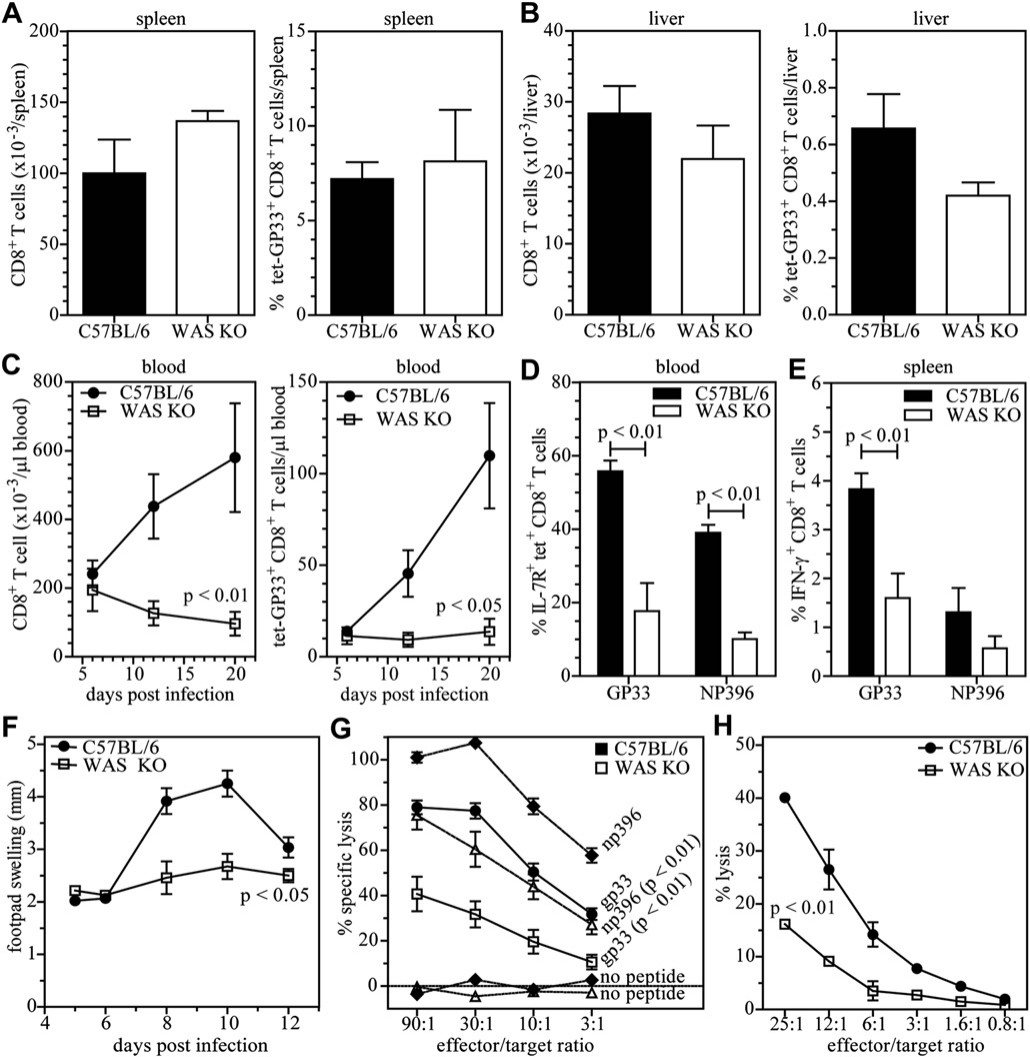
Impaired CD8^+^ T-cell response in patients with WASP deficiency. The virus-specific CD8^+^ T-cell response was analyzed after LCMV infection as the total number of CD8^+^ T cells *(left panel)* or the number of LCMV-specific, GP33-tetramer–positive CD8^+^ T cells *(right panel)* in the spleen **(A)**, liver **(B)**, and blood **(C)**. IL-7R expression was determined on virus-specific CD8^+^ T cells **(D)**. IFN-γ expression was analyzed by using FACS after restimulation of *in vivo*–primed (day 6) virus-specific T cells **(E)**. Swelling of the footpad after LCMV infection was analyzed over time **(F)**. Cytotoxicity of CD8^+^ T cells was determined in virus-specific **(F)** and allogeneic **(G)** settings. Data are shown as means ± SEMs in Fig 3, *A* to *C*, and represent 4 to 8 mice (C57BL/6 mice: day 6, n = 5-6; day 12, n = 7-8; day 20, n = 4-5; WAS KO mice: day 6, n = 6; day 12, n = 6; day 20, n = 4); Fig 3, *D*, n = 3, representative of at least 2 independent experiments; Fig 3, *E*, n = 5 to 6; Fig 3, *F*, n = 6 to 8; Fig 3, *G*, n = 6; and Fig 3, *H*, is a representative experiment of 2 independent experiments with a total of n = 4.

To analyze the CD8^+^ response *in vivo* in more detail, we infected wild-type C57BL/6 or WAS KO mice with LCMV and analyzed the virus-specific CD8^+^ T-cell response. Six days after infection, the total number of CD8^+^ T cells and LCMV-specific, GP33 tetramer–positive CD8^+^ T cells in the spleen, liver, and blood was similar between C57BL/6 and WAS KO mice (Fig 3, *A-C*). However, on days 12 and 20 after infection, the numbers of total and virus-specific CD8^+^ T cells recovered from blood was markedly reduced in WAS KO mice (Fig 3, *C*), whereas at that time, LCMV had been eliminated in C57BL/6 mice (Fig 1, *C*). WAS KO mice also mounted CD8^+^ T-cell responses specific for the immunodominant epitope of the LCMV nucleoprotein (NP396), but although reduced compared with values seen in C57BL/6 mice, this did not reach statistical significance (see Fig E1 in this article's Online Repository at www.jacionline.org). A typical CD8^+^ T-cell response will peak around day 8 after infection, after which only a small subset of CD8^+^ T cells will survive and develop into memory T cells. This subset can be identified by IL-7 receptor (IL-7R) expression.^39,41,42^

We analyzed the expression of IL-7R on GP33 and NP396 tetramer–positive cells at day 8 after infection and found fewer IL-7R^+^ virus–specific CD8^+^ T cells in WAS KO mice (Fig 3, *D*). We then analyzed the function of virus-specific CD8^+^ T cells in more detail and found that splenic WASP-deficient CD8^+^ T cells isolated at day 6 after infection were impaired in their ability to produce intracellular IFN-γ after restimulation *in vitro* with GP33 and NP396 peptides (Fig 3, *E*). Next, we injected LCMV in the footpads of mice because footpad swelling is dependent on viral titer, CD8^+^ T-cell infiltration, and CD8^+^ T-cell cytotoxicity^43^ and found that WAS KO animals exhibited a significantly diminished response (Fig 3, *F*). Finally, we tested the ability of WAS KO CD8^+^ T cells to specifically lyse target cells *in vitro*. T cells were collected at day 6 after infection when antigen-specific cell numbers were equivalent between WAS KO and C57BL/6 mice. WAS KO CD8^+^ T cells showed reduced cytotoxicity to cells presenting the virus-specific GP33 and NP396 epitopes (Fig 3, *G*). To investigate whether this was due to intrinsic dysfunction of WAS KO CD8^+^ T cells, we analyzed the ability of WAS KO CD8^+^ T cells to lyse allogeneic BALB/c splenocytes and observed that WAS KO CD8^+^ T cells also showed reduced cytotoxicity in this nonviral setting (Fig 3, *H*).

Overall, these findings suggest an intrinsic cytotoxic dysfunction of WAS KO CD8^+^ T cells but also a more complex disruption to priming and long-term survival after antigen challenge.

### Impaired priming of CD8^+^ T cells

**Fig 4:**
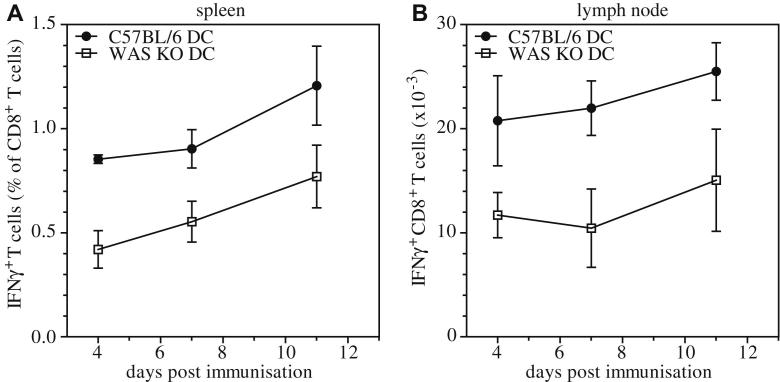
Impaired CD8^+^ T-cell priming. IFN-γ expression of CD8^+^ T cells isolated from spleens **(A)** and lymph nodes **(B)** was determined after *in vivo* priming by ovalbumin-pulsed DCs and subsequent *in vitro* restimulation with ovalbumin peptide. Data are shown as means ± SEMs (day 4, n = 3; day 7, n = 3; day 11, n = 3).

**Fig 5:**
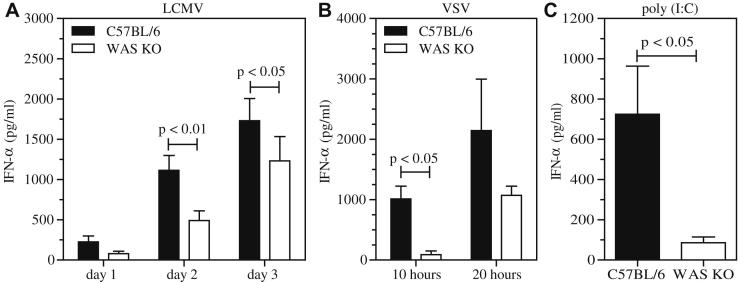
WASP deficiency leads to a reduced IFN-α response. Mice were infected with 200 pfu of the LCMV strain WE **(A)**, 2 × 10^6^ pfu VSV **(B)**, or 200 μg of Poly(I:C) **(C)**, and IFN-α levels were measured in serum at the indicated time points. Data are shown as means ± SEMs (LCMV, n = 6-11; VSV, n = 3; Poly[I:C], n = 6). Serum was taken 3 hours after injection.

Because T cells require antigen-specific stimulation by DCs for optimal priming, we investigated the contribution of defective DC-mediated T-cell priming to abnormal CD8^+^ T-cell responses. We adoptively transferred bone marrow–derived WAS KO DCs pulsed with ovalbumin into wild-type C57BL/6 recipients and analyzed the antigen-specific IFN-γ response. Both in spleens and draining lymph nodes, we observed reduced numbers of IFN-γ–producing wild-type CD8^+^ T cells in response to secondary challenge with ovalbumin (Fig 4, *A* and *B*), suggesting that defective priming by DCs at least in part contributes to defective function of WAS KO CD8^+^ T cells. Priming of virus-specific CD8^+^ T cells is also strongly dependent on IFN-Is, acting either directly on the CD8^+^ T cells or by maturing DCs necessary for antiviral T-cell immunity.^44,45^ Accordingly, we analyzed the IFN-I response in WAS KO mice after infection with LCMV. Induction of serum IFN-α was significantly abrogated in WAS KO mice in response to LCMV infection (Fig 5, *A*). Similarly, when we infected mice with VSV or administered the nonviral, nonreplicating, IFN-I stimulator Toll-like receptor (TLR) 3/RIG-I ligand Poly(I:C) *in vivo*, WAS KO mice exhibited a markedly diminished IFN-α response (Fig 5, *B* and *C*). These findings indicate a general reduction in stimulated IFN-I production *in vivo* in the absence of WASP expression.

### Decreased expression of IFN-I by DCs

**Fig E2:**
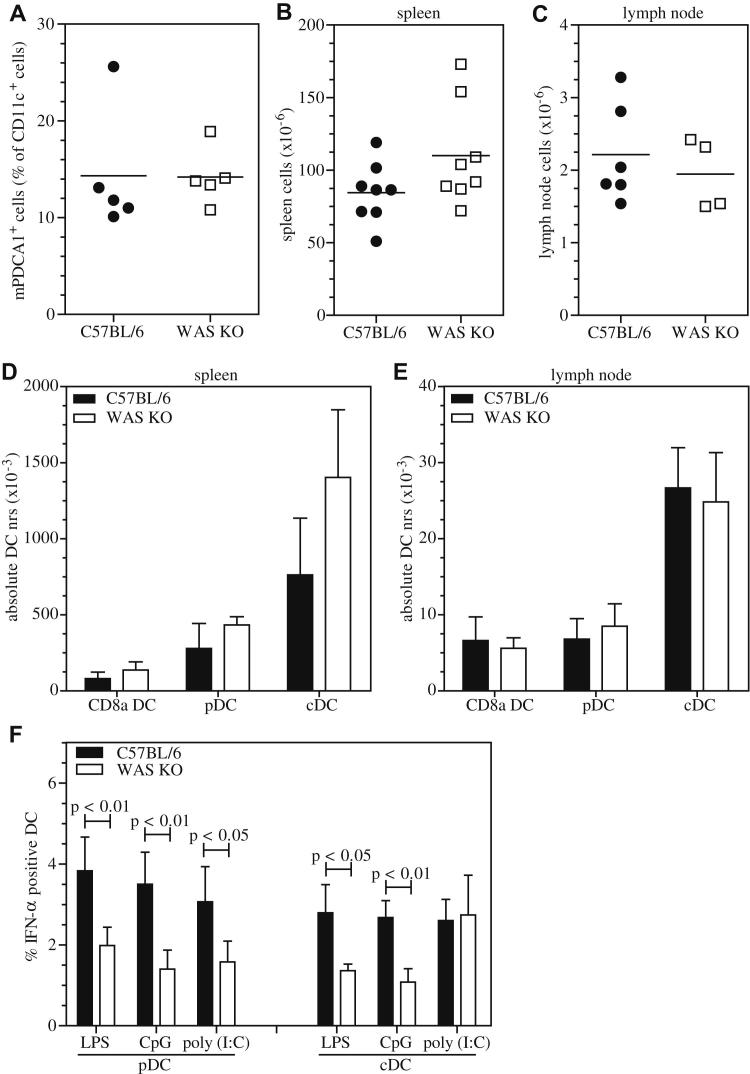
Total number of leukocytes in lymphoid tissue. The number of splenic CD11c^+^mPDCA1^+^ pDCs **(A)** and the total number of leukocytes was determined in spleens **(B)** and lymph nodes **(C)**. DC subsets were quantified in spleens **(D)** and lymph nodes **(E)**. IFN-α expression was determined by means of intracellular flow cytometry after stimulation of *ex vivo*–isolated DC subsets **(F)**. Symbols in Fig E2, *A* to *C*, represent individual mice, and the *line* indicates the mean. Data in Fig E2, *D* to *F*, are expressed as means ± SEMs of n = 4. Fig E2, *F*, LPS and CpG, n = 7; Poly(I:C), n = 4.

**Fig 6:**
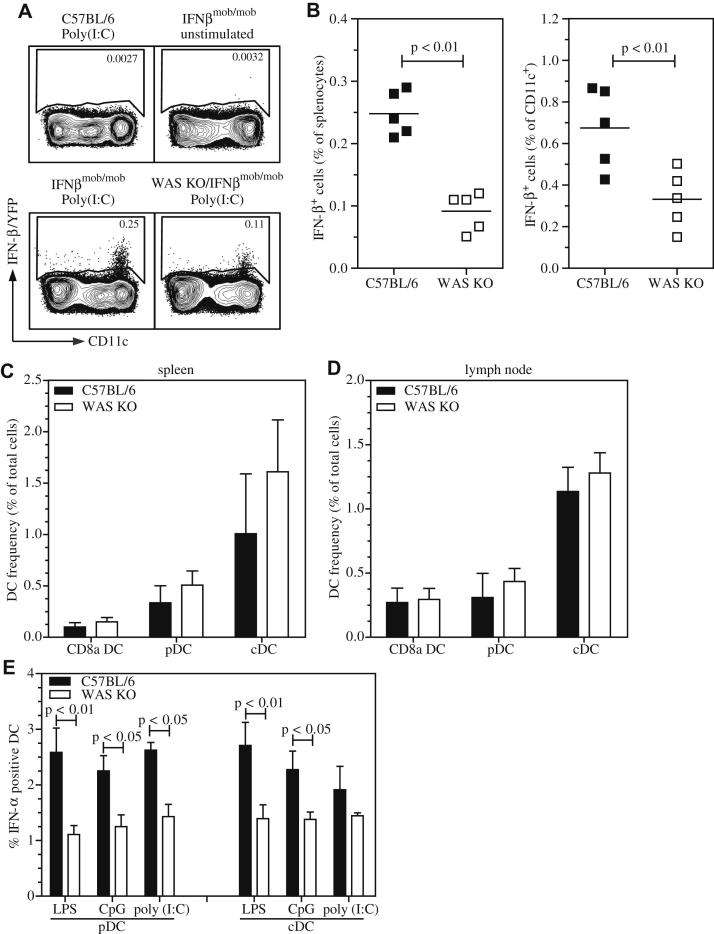
Reduced IFN-I response by DCs. IFN-β/YFP reporter mice were challenged with Poly(I:C), and splenic IFN-β expression was analyzed by using FACS. Representative FACS plots of IFN-β expression by CD11c^+^ DCs after gating for live CD3^−^CD19^−^ cells is shown **(A)**. Also, quantification is shown in as the percentage of all splenocytes *(left)* or as the percentage of CD11c^+^ cells (*right*; **B**). Frequency of DC populations was determined in spleens **(C)** and lymph nodes **(D)**. CD8α^+^ DCs, cDCs, and pDCs were identified as CD11c^+^CD8α^+^CD11b^−^B220^−^ cells, CD11c^+^CD8α^−^CD11b^+^B220^−^ cells, and CD11c^+^CD11b^−^B220^+^ cells, respectively. IFN-α expression was determined by using intracellular FACS of *in vitro*–cultured DC subsets **(E)**. Symbols in Fig 6, *B*, represent individual mice, and the *line* is the mean. Data in Fig 6, *C* to *E*, are expressed as means ± SEMs of (Fig 6, *C* and *D*) n = 4. Fig 6, *E*, LPS and CpG, n = 7; Poly(I:C), n = 4.

To investigate which cells were responsible for the defective production of IFN-Is, we made use of the IFN-β reporter–knock-in mouse, in which yellow fluorescent protein (YFP) expression is bicistronically linked to expression of IFN-β of the endogenous *ifnb* locus, so that IFN-β–producing cells can easily be identified by using YFP expression.^46^ These IFNβ^mob/mob^ mice were crossed with WAS KO mice and challenged with Poly(I:C). As expected, we found that in the absence of WASP, IFN-β/YFP expression was reduced in splenocytes and that this was restricted to CD11c^+^ cells (Fig 6, *A* and *B*). To verify that this was not caused by an overall reduction in the number of DCs in WAS KO mice, we analyzed the proportion of conventional migratory (cDCs; CD11c^+^CD11b^+^CD8α^−^B220^−^), conventional CD8α^+^ (CD11c^+^CD11b^−^CD8α^+^B220^−^), and plasmacytoid (pDCs; CD11c^+^CD11b^−^B220^+^ or CD11c^+^mPDCA1^+^) DC subsets. As expected from previous reports,^14,47^ we did not observe significant differences between distinct subsets in spleens or lymph nodes in C57BL/6 or WAS KO mice (Fig 6, *C* and *D*, and see Fig E2, *A*, in this article's Online Repository at www.jacionline.org). Total splenocyte and lymph node cell numbers in WAS KO and C57BL/6 mice were comparable, as were absolute cell counts of DC subsets (see Fig E2, *B-E*). Finally, we tested whether the impaired production of IFN-I observed *in vivo* reflected an intrinsic deficiency of DCs in the absence of WASP. Both pDCs and cDCs showed a reduced IFN-α response when stimulated with Poly(I:C), CpG, and LPS *in vitro* (Fig 6, *E*). Similarly, when we used *ex vivo*–isolated splenic CD11c^+^ cells, a similar deficiency in IFN-α production in response to Poly(I:C), LPS, and CpG was observed (see Fig E2, *F*). These findings show that WAS KO DCs are intrinsically compromised in their ability to secrete IFN-I.

## Discussion

Patients with WAS have recurrent viral infections,^3,4^ but relatively little is known about the mechanistic role of WASP in antiviral immunity. We found that WAS KO mice did not clear LCMV infection and had exaggerated immunopathology. One possibility is that there was reduced homing of inflammatory cells to the sites of infection.^9^ However, we observed a persistent infiltration of CD4^+^ and CD8^+^ T cells in the liver after viral infection, suggesting that reduced viral clearance is not primarily the result of defective CD8^+^ T-cell migration. In chimeric or transgenic RIP-GP/WAS KO mice, we observed T-cell infiltration around the islets of Langerhans, which is typically associated with the onset of diabetes. Both in antigen-specific and allogeneic settings, we observed reduced cytotoxic function of WAS KO CD8^+^ T cells. Overall, these findings indicate that there are intrinsic defects of cytotoxicity in WAS KO CD8^+^ T cells and that they play a significant role in the control of viral infection *in vivo*. These findings are also in line with a recent report showing that cytotoxicity is reduced in human WASP-deficient CD8^+^ T cells and that WASP is required for delivery and polarization of the lytic granules toward the center of the immunologic synapse.^29^ Similar to cytotoxic T cells, impaired lytic activity of natural killer (NK) cells in patients with WAS has also been reported.^48^ In addition, defective NK cell function can result from impaired DC priming,^49^ but the role of NK cells in LCMV-mediated immunity is expected to be limited because depletion of NK cells improves CD8^+^ T-cell immunity.^50^ Impaired immunologic synapse formation is likely to contribute to defective CD8^+^ T-cell function.^14^ Dependence on WASP for DC-mediated priming has previously also been demonstrated for CD4^+^ T cells and NK cells, in which WASP was shown to be required for formation of an activating immunologic synapse.^6,13,49^ Perhaps most strikingly, although at early time points the CD8^+^ T-cell response to LCMV appears relatively normal, it is poorly sustained compared with that observed in normal mice with fewer IL-7R^+^ cells, marking a reduction in survival and memory CD8^+^ T-cell development. It therefore appears that WASP deficiency not only intrinsically impairs the function of CD8^+^ T cells, as shown by reduced cytotoxicity and IFN-γ production, but also results in abrogated survival or expansion. This might help explain the progressive immunodeficiency observed in patients with WAS as a consequence of accelerated exhaustion.

IFN-Is are crucial for the control of LCMV replication. In complete absence of IFN-Is, no detectable virus-specific CD8^+^ T-cell response is mounted.^36^ Ligation of host pattern-recognition receptors, such as the cytoplasmic helicase RIG-I family and TLR3, TLR7, and TLR9, triggers activation of the transcription factor interferon regulatory factor 7, which translocates to the nucleus, where it promotes IFN-I production.^37,51^ IFN-Is are normally induced at early time points after viral infection and are therefore critical for control of replication and establishment of a definitive immunologic clearance. WAS KO animals demonstrated reduced IFN-I production by DCs after LCMV and VSV infections, as well as after nonviral stimulation of TLR3 and TLR9. Normal pDCs are known for their ability to quickly produce large amounts of IFN-α in response to viral infection or TLR9 ligation. Depletion of pDCs abrogates virus induced IFN-α production and exacerbates virus-induced immunopathology, including diminished CD8^+^ T-cell responses.^52,53^ Normally, a rapid expansion of splenic IFN-I–producing pDCs can be observed in response to LCMV infection.^54^ There were no differences in the frequency of pDCs in WAS KO mice in the steady state, but on stimulation with viral or TLR ligands, WAS KO pDCs showed significantly reduced ability to produce IFN-Is both *in vitro* and *in vivo*. The expansion of virus-specific CD8^+^ T cells has been shown to be strongly dependent on IFN-Is, acting directly on CD8^+^ T cells to promote survival during antigen-driven proliferation and subsequent establishment of memory.^34,43,44,55^ Furthermore, IFN-Is play a key role in enhancing the maturation and activation of DCs.^45^ It therefore seems likely that a reduced IFN-I response in WAS KO mice contributes to the weakened antiviral CD8^+^ T-cell response by directly affecting CD8^+^ T-cell function, influencing the activation of DCs, or their combination. pDC activation by inducible natural killer T cells has been reported to play an important role in control of LCMV infection through stimulation of IFN-I production.^56^ Patients with WAS and WAS KO mice have impaired homeostasis and function of iNKT cells, and therefore it is interesting to speculate that there might be a mechanistic link.^57,58^ Further studies will be required to determine whether defective iNKT cell function affects pDC function in WAS KO model systems.

In conclusion, we have shown that WASP is required to mount a protective antiviral immune response in an *in vivo* model of persistence-prone LCMV infection. In the absence of WASP, a markedly diminished CD8^+^ T-cell response is induced, which most likely is the combination of intrinsic dysfunction of WASP-deficient CD8^+^ T cells and impaired priming and maintenance by IFN-I–producing DCs. This also raises the possibility that IFN-I therapy might be useful for refractory or chronic viral infections in patients with WAS.

**Key messages**

• WASP-deficient mice show reduced viral clearance and enhanced virus-induced immunopathology.
• Priming and effector function of CD8^+^ T cells is impaired in patients with WASP deficiency.
• IFN-I production by DCs is reduced in the absence of WASP.
